# FPGA Implementation of an Efficient FFT Processor for FMCW Radar Signal Processing

**DOI:** 10.3390/s21196443

**Published:** 2021-09-27

**Authors:** Jinmoo Heo, Yongchul Jung, Seongjoo Lee, Yunho Jung

**Affiliations:** 1 Department of Smart Air Mobility, Korea Aerospace University, Goyang-si 10540, Korea; jmz416@kau.kr; 2 Korea Electronics Technology Institute (KETI), Seongnam 13509, Korea; ycjung@keti.re.kr; 3 Department of Information and Communication Engineering and Convergence Engineering for Intelligent Drone, Sejong University, Seoul 05006, Korea; seongjoo@sejong.ac.kr; 4 School of Electronics and Information Engineering, Korea Aerospace University, Goyang-si 10540, Korea

**Keywords:** fast Fourier transform (FFT), memory-based FFT architecture, frequency modulated continuous wave (FMCW) radar, field-programmable gate array (FPGA)

## Abstract

This paper presents the design and implementation results of an efficient fast Fourier transform (FFT) processor for frequency-modulated continuous wave (FMCW) radar signal processing. The proposed FFT processor is designed with a memory-based FFT architecture and supports variable lengths from 64 to 4096. Moreover, it is designed with a floating-point operator to prevent the performance degradation of fixed-point operators. FMCW radar signal processing requires windowing operations to increase the target detection rate by reducing clutter side lobes, magnitude calculation operations based on the FFT results to detect the target, and accumulation operations to improve the detection performance of the target. In addition, in some applications such as the measurement of vital signs, the phase of the FFT result has to be calculated. In general, only the FFT is implemented in the hardware, and the other FMCW radar signal processing is performed in the software. The proposed FFT processor implements not only the FFT, but also windowing, accumulation, and magnitude/phase calculations in the hardware. Therefore, compared with a processor implementing only the FFT, the proposed FFT processor uses 1.69 times the hardware resources but achieves an execution time 7.32 times shorter.

## 1. Introduction

Recently, various types of sensors (passive infrared (PIR), ultrasonic, cameras, and lidar) have been used for target detection [1,2,3,4] but they all have weaknesses. PIR sensors cannot detect stationary targets or multiple targets, and if warm air is injected, a false-alarm detection may occur [1]. Ultrasonic sensors have trouble detecting targets at a distance greater than 5 m, and their angular resolution is poor compared with that of other sensors [2]. Camera sensors are less effective in the dark or in the presence of obstacles, and they require high-performance hardware due to onerous computational signal processing, which also has serious privacy issues [1,3]. Finally, Lidar sensors are limited by their high cost and susceptibility to weather conditions [4].

Unlike these types of sensors, radar sensors have the advantages of not being affected by harsh environmental conditions, such as light and weather, and of being able to measure the range, velocity, and angle of a target directly. It can detect stationary or moving objects and can detect multiple targets simultaneously. Therefore, it is free from privacy issues. Radar sensors can also measure small movements such as breathing and heart rate for vital-sign monitoring and tracking gestures and gait [1]. Because of these strengths, radar sensors are used in industrial machinery, drones, automobiles, and wearable devices [5,6,7,8,9].

Recently, [10] developed a method for detecting a human subject by investigating physical characteristics using Doppler radar. The trained support vector machine (SVM) had an accuracy of 96%. In addition, [11] a method of simultaneously performing target classification and estimating movement direction showed an identification accuracy of 85% even for newly acquired data by using the “you only look once (YOLO)” scheme. In [12], a compact radar system for autonomous walking for the visually impaired and blind was developed. By integrating a Tx/Rx circuit board with a radar antenna, the whole radar system was miniaturized.

Radar can be broadly classified into pulse or frequency-modulated continuous wave (FMCW) radar, the latter of which is simple to implement and has received increasing attention [13]. This radar systems can be divided into slow- and fast-ramp FMCW according to the transmission waveform used. Slow-ramp FMCW uses a triangle-shaped transmission waveform and a pairing technique to extract the range and velocity of the target. However, slow-ramp FMCW suffers from a serious disadvantage in that a ghost target appears when extracting the target’s range and velocity. Therefore, fast-ramp FMCW radar systems are more widely used. These use a sawtooth transmission waveform to extract the target’s range and velocity using a two-dimensional fast Fourier transform (2D FFT) [5,8].

**Figure 1:**
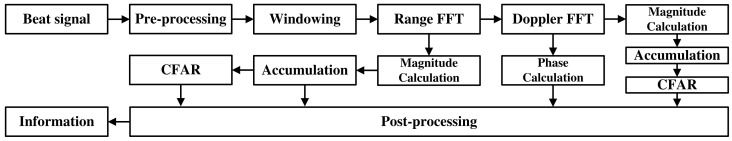
Block diagram of FMCW radar signal processing.

Figure 1 shows a block diagram of typical fast-ramp FMCW radar signal processing for target detection. The received beat signal is digitized in the pre-processing step and the DC component is removed. If a low-reflectance target exists, it may not be detected because of the relatively strong side lobe of the clutter, which is reduced by applying a windowing function before the FFT. The 2D FFT is then applied to extract the range and velocity. The 2D FFT suboperations are called the range FFT and the Doppler FFT, and the result is called a range-Doppler map (RDM). Range FFT and Doppler FFT lengths are parameters that determine the maximum detection range and Doppler resolution. Therefore, FFT processors must support variable lengths because the length used depends on the performance required for each application [14].

FFT processors are generally designed and implemented using a fixed-point format because of their simplicity. However, because fixed-point formats have a limited number representation range, implementing the FFT with a fixed-point operator requires adjusting the result according to the possible representation. Therefore, fixed-point FFT processors offer poor FFT performance because of quantization noise (Q-noise). In FMCW radar, Q-noise accumulates because the FFT is repeatedly performed during signal processing. Therefore, many studies have proposed designing and implementing the FFT processor using a floating-point operator [15,16,17,18,19].

After 2D FFT, it is common to conduct constant false alarm rate (CFAR) detection. The range and velocity of a target are measured by changing the threshold depending on the local average noise power. The accumulation of magnitude components improves detection performance [20,21,22,23,24]. Therefore, it is necessary to calculate and accumulate the magnitude component of the FFT result. In addition, the phase component of the FFT result is often used to acquire various vital signs such as respiration and heart rate [25,26]. Therefore, it is also necessary to calculate the phase component of the FFT result.

Because FFT computations are the most resource-intensive among these, an optimized hardware implementation is required. The hardware architecture of FFT processors can be divided into two types: pipelined FFTs and memory-based FFTs. Memory-based FFTs are also called “in-place” or “iterative” FFTs [27]. In long FFT processors, pipelined FFT structures consume a lot of area, so memory-based FFT structures are preferred [28,29,30,31,32]. Furthermore, given the computational speed and variable length of the transformations, it is generally appropriate to use a mixed-radix butterfly unit with a mixture of radix-4 and radix-2 [28].

In this paper, we propose an FFT processor hardware structure supporting a variable length of 64–4096 and windowing, magnitude/phase calculation, and accumulation operations. We also present the results of an FPGA-based implementation of the proposed processor. Our results show that the proposed FFT processor can carry out the signal processing required for FMCW radar systems, reduce computation times, and achieve a high signal-to-quantification-noise ratio (SQNR) performance by using a floating-point operator.

The rest of this paper is organized as follows. Section 2 reviews the FMCW radar signal-processing algorithm. Section 3 describes the hardware architecture of the proposed FFT processor. Section 4 presents the implementation results. Finally, Section 5 presents our conclusions.

## 2. FMCW Radar Algorithm

### 2.1. Measuring Range and Velocity in FMCW Radar

FMCW radar employs a transmission waveform the frequency of which varies linearly with time. This waveform can be either triangular or sawtooth. It is challenging to use FMCW radar based on the triangular transmission waveform in multi-target scenarios because it is difficult to remove the ghost target signal. Therefore, FMCW radar systems with sawtooth transmission waveforms are commonly used. The frequency of the sawtooth waveform is defined by Equation (1).

(1) $$f\left(t\right)=f_{c}+\frac{B}{T}t,$$

where $f_{c}$ is the carrier frequency; *B* is the bandwidth; *T* is the period; and *t* is time. For convenience, B/T is replaced by α. The instantaneous phase of the transmission waveform can be obtained by integrating the frequency of the transmission waveform with respect to time *t*, as in Equation (2).

(2) $$\varphi \left(t\right)=2\pi \int_{0}^{t}f\left(t\right)dt=2\pi \int_{0}^{t}\left(f_{c}+\alpha t\right)dt=2\pi \left(f_{c}t+\frac{\alpha t^{2}}{2}\right)$$

Here, $t=nT+t_{s}$, and $t_{s}$ is the time between 0 and *T*. If the initial phase of the transmission signal is $\varphi_{0}$ and the amplitude is *A*, the transmission waveform of the first chirp is given by Equation (3).

(3) $$s_{Tx}\left(t\right)=A\;cos\left(2\pi \left(f_{c}t+\frac{\alpha t^{2}}{2}\right)+\varphi_{0}\right)$$

By substituting $t=nT+t_{s}$ into Equation (3), the *n*th transmission signal can be obtained as shown in Equation (4).

(4) $$s_{Tx}\left(n,t_{s}\right)=A\;cos\left(2\pi \left(f_{c}\left(nT+t_{s}\right)+\frac{\alpha {\left(nT+Ts\right)}^{2}}{2}\right)+\varphi_{0}\right)$$

After the delay time τ, the received signal can be expressed as in Equations (5) and (6).

(5) $$s_{Rx}\left(n,t_{s}\right)=A^{\prime }\;cos\left(2\pi \left(f_{c}\left(nT+t_{s}-\tau \right)+\frac{\alpha {\left(nT+Ts-\tau \right)}^{2}}{2}\right)+\varphi_{0}\right)$$

(6) $$\tau =\frac{2(R+vt)}{c}=\frac{2(R+v\left(nT+t_{s}\right))}{c}$$

Here, $A^{\prime }$ is the amplitude of the received signal; *R* is the range of the target; *v* is the velocity of the target, and *c* is the speed of light.

Demodulation is performed by multiplying the received signal reflected from the target and the transmitted signal as shown in Equation (7).

(7) $$s_{M}\left(n,t_{s}\right)=s_{Tx}\left(n,t_{s}\right)\times s_{Rx}\left(n,t_{s}\right)$$

The phase component of the demodulated signal $s_{M}\left(n,t_{s}\right)$ is in the form of a sum of cosine terms, and the high-frequency signal is removed by a low-pass filter. Thus, the in-phase components of the signal can be arranged as shown in Equation (8).

(8) $$\begin{array}{cc} \; & s_{M-I}\left(n,t_{s}\right)=\frac{C}{2}\;cos\left(2\pi \left(f_{c}\tau +\alpha \tau t_{s}-\frac{\alpha \tau^{2}}{2}\right)\right)\\ \; & =\frac{C}{2}\;cos(2\pi (f_{c}\left(\frac{2(R+v\left(nT+t_{s}\right))}{c}\right)+\alpha t_{s}\left(\frac{2(R+v\left(nT+t_{s}\right))}{c}\right)\\ \; & -\frac{\alpha }{2}{\left(\frac{2(R+v\left(nT+t_{s}\right))}{c}\right)}^{2})), \end{array}$$

where *C* is the product of *A* and $A^{\prime }$. Equation (9) can be derived by expanding Equation (8).

(9) $$\begin{array}{cc} \; & s_{M-I}\left(n,t_{s}\right)=\frac{C}{2}\;cos(2\pi (\left(\frac{2\alpha R}{c}+\frac{2f_{c}v}{c}+\frac{2\alpha vnT}{c}-\frac{4\alpha Rv}{c^{2}}-\frac{4\alpha nTv^{2}}{c^{2}}\right)t_{s}\\ \; & +\left(\frac{2f_{c}v}{c}-\frac{4\alpha Rv}{c^{2}}\right)nT+\frac{2f_{c}R}{c}+\frac{2\alpha v{t_{s}}^{2}}{c}-\frac{2\alpha R^{2}}{c^{2}}-\frac{2\alpha v^{2}n^{2}T^{2}}{c^{2}}-\frac{2\alpha v^{2}{t_{s}}^{2}}{c^{2}})) \end{array}$$

In Equation (9), c is very large, so $1/c^{2}$ terms are negligible. Moreover, $2f_{c}v/c$ and 2αvnT/c are very small compared to 2αR/c and can be ignored. If the same approach is applid to $2\alpha v{t_{s}}^{2}/c$, Equation (9) can be approximated by the expression for $s_{M-I}$ shown in Equation (10). Through the same process, the quadrature components can be approximated by Equation (11).

(10) $$s_{M-I}\left(n,t_{s}\right)=\frac{C}{2}\;cos\left(2\pi \left(\frac{2\alpha R}{c}t_{s}+\frac{2f_{c}v}{c}nT+\frac{2f_{c}R}{c}\right)\right)$$

(11) $$s_{M-Q}\left(n,t_{s}\right)=\frac{C}{2}\;sin\left(2\pi \left(\frac{2\alpha R}{c}t_{s}+\frac{2f_{c}v}{c}nT+\frac{2f_{c}R}{c}\right)\right)$$

The range to the target *R* and the beat frequency $f_{b}$ can be defined as in Equations (12) and (13).

(12) $$R=\frac{c\tau }{2}=\frac{cf_{b}}{2\alpha }=f_{b}\times \Delta R\times T$$

(13) $$f_{b}=\frac{2\alpha R}{c}$$

The frequency of the received signal reflected by the moving target can be defined as shown in Equation (14) by considering the Doppler effect.

(14) $$f_{r}=\left(\frac{1+v/c}{1-v/c}\right)f_{t}$$

Here, $f_{r}$ is the reception frequency, and $f_{t}$ is the transmission frequency. Equation (14) can be transformed into Equation (15) using the binomial series.

(15) $$f_{r}=\left(1+\frac{v}{c}\right)\left(1+\left(\frac{v}{c}\right)+{\left(\frac{v}{c}\right)}^{2}+\cdots \right)f_{t}=\left(1+2\frac{v}{c}+2{\left(\frac{v}{c}\right)}^{2}+\cdots \right)f_{t}$$

Because the speed of light *c* is very large, the higher-order terms can be removed to obtain Equation (16).

(16) $$f_{r}=\left(1+2\left(\frac{v}{c}\right)\right)f_{t}=f_{t}+2\left(\frac{v}{c}\right)f_{t}$$

The Doppler frequency is defined as $f_{d}=2vf_{c}/c$. By substituting $f_{b}$ and $f_{d}$ in Equations (10) and (11), Equations (17) and (18) are obtained.

(17) $$s_{M-I}\left(n,t_{s}\right)=\frac{C}{2}\;cos\left(2\pi \left(f_{b}t_{s}+f_{d}nT+\frac{2f_{c}R}{c}\right)\right)$$

(18) $$s_{M-Q}\left(n,t_{s}\right)=\frac{C}{2}\;sin\left(2\pi \left(f_{b}t_{s}+f_{d}nT+\frac{2f_{c}R}{c}\right)\right)$$

Equation (19) can be derived by expressing the in-phase and quadrature components in exponential functions using Euler’s formula.

(19) $$s_{M}\left(n,t_{s}\right)=\frac{C}{2}\;exp\left(j2\pi \left(f_{b}t_{s}+f_{d}nT+\frac{2f_{c}R}{c}\right)\right)$$

The beat frequency can be obtained by performing the discrete Fourier transform (DFT) on the expression of $s_{M}\left(n,t_{s}\right)$ shown in Equation (19) for one chirp, that is, $t_{s}$. Using Equation (12), we can obtain the range to the target from the beat frequency. In addition, the Doppler frequency can be obtained by performing the DFT on the frequency change of the signal for several chirps, that is, nT. Because $f_{d}=2vf_{c}/c$, we can use the Doppler frequency to calculate the velocity of the target.

If the number of samples in the range direction, i.e., the range FFT length, is defined as *M*, the sampling interval becomes T/M, and thus the sampling frequency $F_{s}$ is given by Equation (20). Furthermore, the relationship between the maximum detection range $R_{max}$ and *M* can be derived from Equations (21) and (22).

(20) $$F_{s}=\frac{M}{T}$$

(21) $$R_{max}=f_{b}T\Delta R=\frac{F_{s}}{2}T\Delta R=\frac{M}{2}\Delta R=\frac{cM}{4B}$$

(22) $$M=\frac{4BR_{max}}{c}$$

Moreover, if we define the number of chirps, i.e., the Doppler FFT length, as *N*, then the sampling frequency is 1/T, and $\Delta f_{D}$ can be derived as in Equation (23).

(23) $$\Delta f_{D}=\frac{1}{NT}=\frac{2}{\lambda }\Delta v$$

(24) $$\Delta v=\frac{\lambda }{2NT}$$

(25) $$N=\frac{\lambda }{2T\Delta v}$$

Here, Δv is the Doppler resolution. Equations (22) and (25) confirm that the range FFT and Doppler FFT lengths are essential parameters for determining the maximum detection range and Doppler resolution, respectively. Depending on the radar application, the maximum detection range and Doppler resolution requirements vary. Therefore, the FFT processor should ideally support variable lengths.

### 2.2. CFAR Algorithm

The simplest way to detect the range and velocity of a target in an FMCW radar system is to set a constant threshold. The detection algorithm then compares the magnitude component of the FFT result to this threshold. However, the average noise power varies with time. This is because various parameters of the environment where the radar operates, such as temperature and humidity, are not constant. Therefore, the false alarm detection rate can be very high while using a constant threshold. False alarms directly affect system performance by wasting radar resources owing to continuous detection.

The CFAR algorithm is widely used to reduce the false alarm rate in radar systems. The CFAR algorithm does not maintain the threshold constant but instead adjusts it according to the average noise power. The basic CFAR algorithm proceeds as follows. (1) The magnitude component of the FFT result is calculated. (2) The signal for which it needs to be determined whether it is a target or not is called a test signal. The average local noise power is generated by the surrounding signals. (3) The algorithm checks if a test signal is a target by comparing it to the threshold generated using the surrounding signals. (4) Finally, steps (2) and (3) are repeated for all signals.

The FMCW radar system should apply CFAR detection using to both the range and Doppler axes directions to extract the range and velocity information from the target. To improve the detection performance, 1D data are generated by accumulating RDMs over the range or Doppler axes directions [23,24]. Therefore, a function to calculate and accumulate the FFT results into a magnitude component is required.

## 3. Hardware Architecture of the Proposed FFT Processor

**Figure 2:**
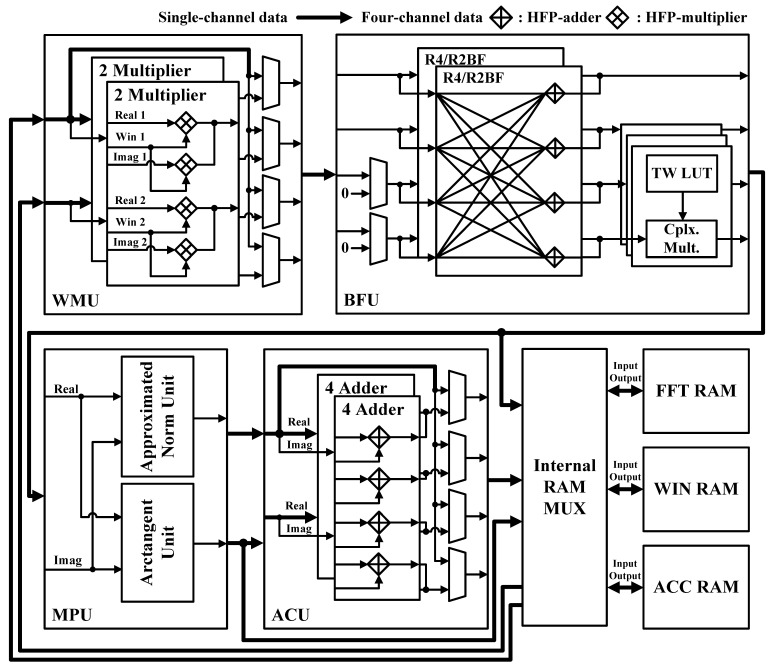
Hardware architecture of the proposed FFT processor.

As shown in Figure 2, the proposed FFT processor consists of a window multiplication unit (WMU), a butterfly unit (BFU), a magnitude/phase calculation unit (MPU), and an accumulation unit (ACU). In addition, it was designed with four channels to reduce execution time. The memory of the processor consists of FFT RAM to store input/output values, WIN RAM to store window coefficient values, and ACC RAM to store accumulated values.

The WMU performs windowing before the FFT operation. The WMU was designed so as to operate by reading from a separate WIN RAM. Therefore, the window coefficients can be changed easily by the user. Windowing is performed on the input data, but no windowing is performed on the intermediate calculated values of the FFT. Therefore, the WMU selectively outputs through a multiplexer (MUX). In addition, because only the real value of the window function is used, eight multipliers are used.

The BFU performs the butterfly operation of the FFT. This unit can perform radix-4/2 butterfly operations for various transform lengths. Because the input comes from four channels, inputs 3 and 4 are set to zero when radix-2 butterfly operations are performed. The intermediate value of the FFT obtained through the BFU is stored in the FFT RAM. Then, the BFU repeatedly performs butterfly operations until the final FFT result is obtained.

The MPU performs an operation that calculates, from the FFT result, the corresponding magnitude and phase components. We implemented it using an algorithm that approximates the magnitude and phase components to reduce the necessary hardware resources. Therefore, we implemented the MPU using only shifters and adders. The algorithms for approximating the magnitude and phase are discussed in detail in Section 3.2.

The ACU accumulates the FFT results. In contrast to windowing, accumulation is performed directly on the FFT results, but not on the intermediate calculated values of the FFT. Therefore, the ACU selectively outputs through a MUX. The accumulation process requires adding the current FFT result to the accumulated value. Thus, the accumulated values are written to, and read from, a separate ACC RAM.

### 3.1. HFP Operation

To measure the range and velocity of a target using the FMCW radar, 2D FFT had to be performed on the input data from ADC. Since 2D FFT increases quantization noise compared to the 1D FFT, essential information may be lost. For example, in the case of a hand gesture recognition radar system, the value of the echo signal was very small because the radar cross section of the human hand is very small [33,34]. If the quantization noise is overlapped and increased by the 2D FFT, important data for a hand gesture with a small echo signal value may be lost. To achieve a reasonable recognition performance, the SQNR of the 2D FFT needed to be large enough.

Table 1 compares the 2D FFT SQNR performance based on a fixed-point and a floating-point operator when the number of bits in the input data ranged from 16 to 28. When the floating-point number system hads 16-bits of data, it was called a half-precision floating-point (HFP) format. As shown in Table 1 , the SQNR degradation occurs seriously in the fixed-point number system, especially when the number of bits in the input data was 16 to 24 for 4096×4096 data. Fixed-point systems did not exhibit significant performance penalties when the bit width was 28 bits; their SQNR performance was close to that of HFP systems.

Table 2 compares the hardware resources used after designing and synthesizing an FFT processor based on either the 28-bit fixed-point operator or the HFP operator. The results showed that the FFT processor, implemented with the HFP operator, used more LUTs than that implemented by the fixed-point operator. Because the Xilinx FPGA’s block RAM (BRAM) is composed of 16-bit units, an FFT processor implemented with a fixed-point operator required twice the BRAM.

The Xilinx FPGA’s DSP consists of fixed bits of the complement multipliers of two and is used to implement multipliers. Because the number of DSP bits is fixed, the DSP is used extensively during multiplications if the bit width is large. Therefore, a processor configured with a fixed-point operator will require approximately three times the DSP capacity. Therefore, it seems preferable to design an FFT processor with an HFP operator from the point of view of FFT performance degradation and the required hardware.

**Figure 3:**
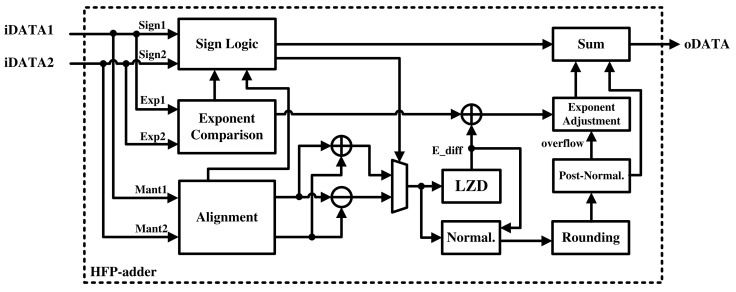
Hardware architecture of the HFP-adder.

A floating-point number consists of a sign, an exponent, and a mantissa and performs operations by treating their components separately. The HFP-adder performs addition by separating the input data into sign, exponent, and mantissa, as shown in Figure 3. The sign is determined using sign logic. If two numbers have the same sign, the sign of the addition result is the same. If the signs of the two numbers are different, the sign of the result must be determined by comparing the numbers’ exponents and mantissas. Finally, sign logic is used to determine the sign of addition and the addition/subtraction of the mantissa.

The exponent of the result is determined in three steps. First, the larger value is selected by comparing the two exponent values. Then, the difference resulting from the mantissa calculation is added. Finally, if overflow occurred in post-normalization, it is adjusted to determine the final exponent. Adjustment ensures that the exponent did not overflow.

The mantissa is determined through a more complex process than that for the previous two components. It is calculated through a process of alignment, operation, normalization, rounding, and post-normalization. First, if the two input values have different exponents, an alignment process is required to match the number of digits. To use only one addition and subtraction operator, we compared two values of the mantissa and swapped them. After matching and swapping, the operation result was added or subtracted according to sign logic.

Then, leading zeros are detected; changes in the exponent value are calculated; and normalization to the floating-point format is performed. The least significant bits (LSBs) lost in this process are used as rounding bits, which are used to perfoem rounding and normalization. If an overflow occurs, normalization is performed again through post-normalization. Finally, the components are combined to generate the final result.

**Figure 4:**
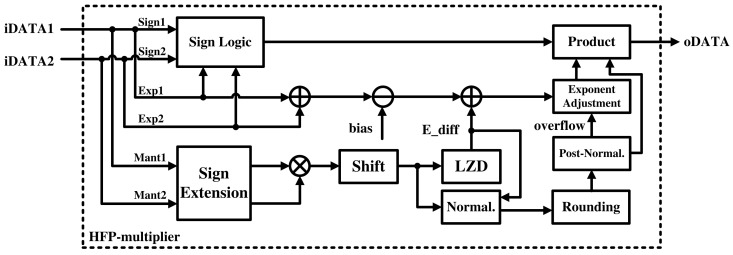
Hardware architecture of the HFP-multiplier.

Similar to the HFP-adder, the HFP-multiplier performs multiplication by separating the input data into sign, exponent, and mantissa, as shown in Figure 4. The sign is determined by an exclusive-OR logic gate. The exponent of the floating-point number system uses a biased notation instead of a two’s complement [35]. Therefore, the HFP-multiplier adds the two exponent values and subtracts the bias values. Then, the difference resulting from the mantissa calculation is added. Finally, the result is adjusted to ensure the exponent does not overflow.

Mantissa calculations are performed in the following order: operation, normalization, rounding, and post-normalization. In contrast to the HFP-adders, the HFP-multipliers do not require alignment processing because multiplication can be conducted for inputs with any number of digits. The bits of the multiplication result are twice those of the operand. Because the number of bits of the multiplication result is too large, we reduce the number by shifting. The LSB that is lost at this point is used to generate three rounding bits.

Subsequently, an HFP-adder-like process follows. The leading zeros are detected and the changes in the exponent value are calculated. Normalization to floating-point format is then performed. The LSBs lost in this process are used as rounding bits. Rounding is then performed using the rounding bits generated in the operation and normalization steps. Again, overflows may occur during rounding; if it does, normalization is performed again through post-normalization. Finally, the components are combined to generate the final result.

### 3.2. Magnitude/Phase Calculation Unit

The MPU is used to calculate the magnitude and phase components of the FFT result. If the magnitude component is calculated using an approximation method, such as that shown in Equation (26), the number of calculations can be efficiently reduced by replacing the multiplication with an addition without significant performance degradation [36].

(26) $$∥x∥=\frac{3}{8}\left(\left|Re(x)\right|+\left|Im(x)\right|\right)+\frac{5}{8}max\left(\left|Re(x)\right|,\left|Im(x)\right|\right)$$

Here, *x* is the FFT result; Re(·) is a function that calculates the real part of an input value; Im(·) is a function that calculates the imaginary part of an input value; and max(a,b) is a function that selects the largest between two given values. In this case, it selects the largest absolute value between the real and imaginary parts.

**Figure 5:**
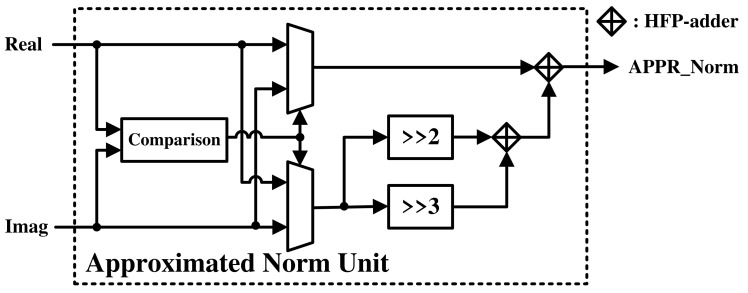
Hardware architecture of the approximated norm unit.

The approximated norm unit was implemented as shown in Figure 5. After comparing the real and imaginary parts, the resulting magnitude is approximated using shifters and adders. A comparison between two floating-point numbers is performed through exponent and mantissa comparisons. The shifter subtracts the exponent by 2 and 3 to make 1/4 and 1/8, respectively. Finally, the numbers are added using HFP-adders, producing the same result as Equation (26).

The calculation of the phase component of the FFT result was implemented using a coordinate rotation digital computer (CORDIC) algorithm. The CORDIC algorithm is an iterative computation method that views a function as a vector in a two-dimensional plane and obtains a converged value through repeated vector rotation. In Equations (27) through (29), if a real value is substituted in $x^{\left(1\right)}$ and an imaginary value is substituted in $y^{\left(1\right)}$ and the operation is repeatedly performed until $y^{\left(i\right)}$ becomes 0, the phase value comes out in $z^{\left(i\right)}$ [37]. Here, $d_{i}=-sign\left(x^{\left(i\right)}\cdot y^{\left(i\right)}\right)$.

(27) $$x^{\left(i+1\right)}=x^{\left(i\right)}-d_{i}y^{\left(i\right)}2^{\left(-i\right)}$$

(28) $$y^{\left(i+1\right)}=y^{\left(i\right)}+d_{i}x^{\left(i\right)}2^{\left(-i\right)}$$

(29) $$z^{\left(i+1\right)}=z^{\left(i\right)}-d_{i}\times arctan\left(2^{\left(-i\right)}\right)$$

**Figure 6:**
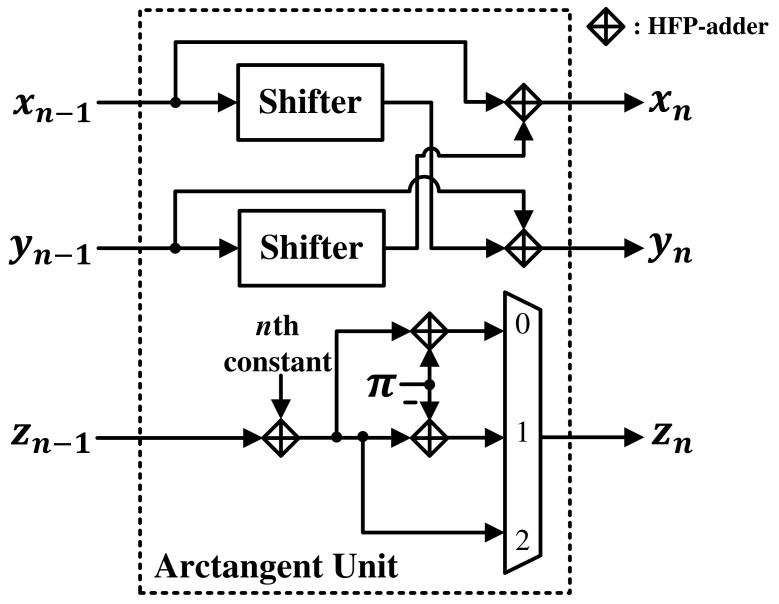
Hardware architecture of the arctangent unit.

The arctangent unit, composed of a shifter, a controller, a MUX, and an adder, was implemented as shown in Figure 6. When implementing CORDIC in a pipeline architecture, units must be used as many times as the number of iterations. Therefore, one unit is used repeatedly to calculate the phasor component. The shifter was implemented so that the exponent could be subtracted from 0 to 12, and the *n*th constant had a value of arctan $\left(2^{\left(-i\right)}\right)$.

## 4. Implementation Results of the Proposed FFT Processor

The proposed FFT processor was designed using hardware description language (HDL) and implemented on a Xilinx Zynq UltraScale+ device-based FPGA platform. As shown in Table 3, the FFT processor was implemented with 10,891 LUTs, 6365 FFs, and 20 DSPs. It used 1.69 times more hardware resources than the BFU, which performed only the FFT operation.

**Figure 7:**
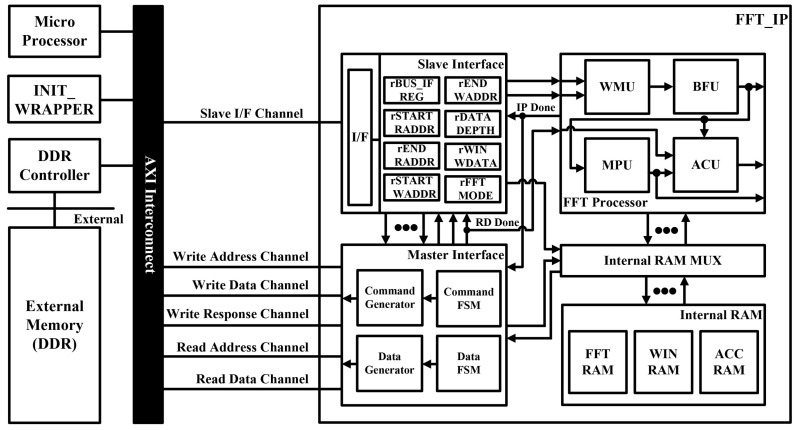
FPGA platform configuration for the verification of the proposed FFT processor.

**Figure 8:**
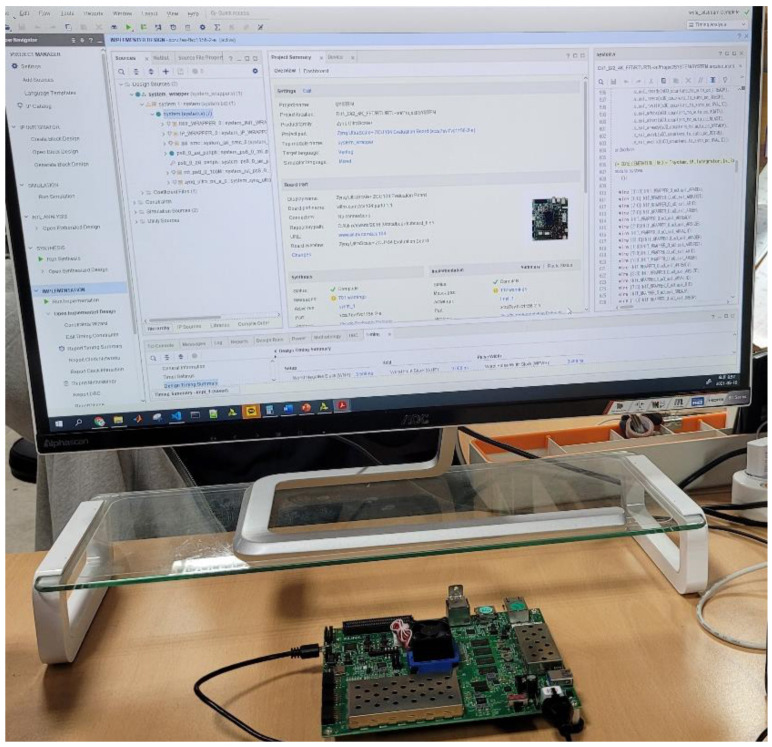
Verification environment using an FPGA platform.

As shown in Figure 7, the proposed FFT processor was configured on the FPGA platform using an advanced extensible interface (AXI) bus interface for verification. Figure 8 shows the verification environment for the FPGA platform. The system structure consisted of an FFT processor, a master interface for data transmission/reception with double data-rate (DDR) memory, a slave interface for communication with a microprocessor (MP), internal RAM and a register that can change the operation mode of the FFT processor. Input data for hardware verification were initialized in DDR memory, and FFT length was set using the MP. When the start signal of the FFT IP was input through the MP, the initial data of the DDR memory was stored in the internal RAM of the FFT IP. After reading all the data, the FFT processor performed the necessary operations. When these operations were completed, the result was stored in DDR memory through the master interface.

Table 4 shows the evaluation results forexecution the time of FMCW radar signal processing, which refers to windowing the input data, performing a 2D FFT, calculating the magnitude/phase components, and accumulating it. To evaluate the speed of the FFT processor, we implemented different versions and measured their execution times across three versions: one using only software, one using dedicated hardware only for the FFT (similar to existing FFT processors), and one using the proposed FFT processor.

When performing radar signal processing with 4096 × 4096 data, implementing only the FFT in the hardware shortened the execution time from 32.97 to 4.54 s compared to than using only software. This corresponded to a 7.26-fold acceleration. Execution time was reduced from 32.97 to 0.62 s when implementing the proposed FFT processor instead of only software. This corresponded to a 53.29-fold acceleration. Compared to implementing only FFT in hardware, the proposed FFT processor accelerated the radar signal processing by 7.32 times.

Table 5 shows a comparison between the hardware resources of the proposed FFT processor and those of an existing FFT processor [38] and Xilinx’s FFT IP [39], both of which were implemented with a memory-based architecture using a floating-point operator. Since the memory-based FFT architecture was implemented based on a single butterfly operator, the effect of the transform length on LUT and FF in FPGA was not significant. Therefore, the normalization for transform length was not applied. Because they only performed the FFT operation, it was more appropriate to consider only the hardware resources of the BFU of the proposed processor. Even though the LUT and FF of [38] were normalized by the bit width, it could be seen that the BFU of the proposed processor required fewer hardware resources. Compared with [39], the BFU of the proposed FFT processor required a similar amount of hardware resources with a similar clock frequency. However, the proposed FFT processor is expected to be much faster than that of [39] for FMCW radar signal processing owing to the integration of the WMU, MPU, and ACU. Therefore, the proposed FFT processor is more efficient than the others when considering the trade-off between hardware resources and execution time.

## 5. Discussion and Conclusions

We developed an FFT processor for FMCW radar signal processing to support variable lengths by applying a mixed-radix algorithm. It also supports windowing, magnitude/phase calculations, and accumulation functions. The processor was implemented using a Xilinx Zynq UltraScale+ device. In our implementation, 10,891 LUTs, 6365 FFs, 10 RAM blocks, and 20 DSPs were used as hardware resources.

Since the general FFT processor only supports FFT operation, it is appropriate to compare it with the BFU of the proposed processor. The Xilinx FFT processor and the BFU of the proposed FFT processor used similar hardware resources. However, the proposed processor required more hardware resources. Comparing the execution time of windowing, 2D FFT, magnitude/phase calculation, and accumulation, the proposed processor significantly shortened it 7.32 times compared to the Xilinx FFT processor.

As mentioned, the proposed FFT processor supported a high SQNR and special functions such as windowing, magnitude/phase calculation, and accumulation. Therefore, it is very efficient for FMCW radar signal processing and can be used for other applications such as wireless communication with orthogonal frequency division multiplexing (OFDM) modulation and voice recognition systems with frequency analysis, which requires a high SQNR and the abovementioned special functions [40,41].

In future work, we will implement a radar signal processor that includes the proposed FFT processor in VLSI. It and will be expected to find wide use in automobiles, drones and wearable devices that require low-cost, llow-power implementation. 

## Figures and Tables

**Table 1:** Comparison of 2D FFT SQNR (dB) of fixed-point and floating-point number systems.

Format	64 × 64	128 × 128	256 × 256	512 × 512
Fixed-point (16 bits)	34	26	22	14
Fixed-point (20 bits)	51	50	45	38
Fixed-point (24 bits)	52	51	51	50
Fixed-point (28 bits)	52	51	51	51
HFP (16 bits)	59	57	57	55
**Format**	**1024 × 1024**	**2048 × 2048**	**4096 × 4096**	
Fixed-point (16 bits)	10	2	0	
Fixed-point (20 bits)	34	26	22	
Fixed-point (24 bits)	45	41	35	
Fixed-point (28 bits)	51	50	50	
HFP (16 bits)	54	53	52	

**Table 2:** Comparison of hardware resources.

Resource	Fixed-Point	HFP
Device	Zynq UltraScale+	Zynq UltraScale+
Bit width	28	16
Radix	2, 4	2, 4
LUT	9846	10,891
FF	7377	6365
BRAM	20	10
DSP	76	20

**Table 3:** Implementation results of the proposed FFT processor.

Block	LUT	FF	DSP
WMU	637	393	8
BFU	6430	3570	12
MPU	2868	1811	0
ACU	956	591	0
Total	10,891	6365	0

**Table 4:** Execution time for FMCW radar signal processing when using the proposed FFT processor.

Data Size	Execution Time (ms)		
	Full SW	FFT Accel.	WMU/FFT/MPU/ACU (Full) Accel.
64 × 64	4.40	1.89	0.61
128 × 128	20.10	6.39	1.20
256 × 256	90.14	23.89	3.00
512 × 512	399.59	94.89	11.16
1024 × 1024	1761.42	373.39	38.22
2048 × 2048	7706.01	1331.16	168.06
4096 × 4096	32,973.46	4537.62	618.78

**Table 5:** Comparison of the proposed FFT processor with those from previous studies.

	[38]	[39]	Proposed	
			BFU	FFT
FPGA	Virtex-4	Zynq UltraScale+	Zynq UltraScale+	Zynq UltraScale+
Architecture	Memory-based	Memory-based	Memory-based	Memory-based
Transform length	1024	4096	64–4096	64–4096
Radix	4	4	2, 4	2, 4
Format	Floating-point	Floating-point	Floating-point	Floating-point
Windowing	-	-	-	O
Mag/Phase	-	-	-	O
Accumulation	-	-	-	O
LUT	24,472	6237	6430	10,891
FF	13,834	3756	3570	6365
Clock freq. (MHz)	100	300	300	300

## References

[1] Cardillo E., Li C., Caddemi A. (2021). Embedded heating, ventilation, and air-conditioning control systems: From traditional technologies toward radar advanced sensing. Rev. Sci. Instrum..

[2] Shao Y., Chen P., Cao T. A grid projection method based on ultrasonic sensor for parking space detection. Proceedings of the IGARSS 2018—2018 IEEE International Geoscience and Remote Sensing Symposium.

[3] Son Y., Heo S.W. A novel multi-target detection algorithm for automotive FMCW radar. Proceedings of the 2018 International Conference on Electronics, Information, and Communication (ICEIC).

[4] Han J., Liao Y., Zhang J., Wang S., Li S. (2018). Target fusion detection of LiDAR and camera based on the improved YOLO algorithm. Mathematics.

[5] Piotrowsky L., Jaeschke T., Kueppers S., Siska J., Pohl N. (2019). Enabling high accuracy distance measurements with FMCW radar sensors. IEEE Trans. Microw. Theory Tech..

[6] Park J., Park S., Kim D.H., Park S.O. (2019). Leakage mitigation in heterodyne FMCW radar for small drone detection with stationary point concentration technique. IEEE Trans. Microw. Theory Tech..

[7] Pérez R., Schubert F., Rasshofer R., Biebl E. Single-frame vulnerable road users classification with a 77 GHz FMCW radar sensor and a convolutional neural network. Proceedings of the 2018 19th International Radar Symposium (IRS).

[8] Zhang Z., Tian Z., Zhou M. (2018). Latern: Dynamic continuous hand gesture recognition using FMCW radar sensor. IEEE Sens. J..

[9] Hyun E., Jin Y.S., Lee J.H. Moving and stationary target detection scheme using coherent integration and subtraction for automotive FMCW radar systems. Proceedings of the 2017 IEEE Radar Conference (RadarConf).

[10] Kim Y., Ha S., Kwon J. (2014). Human detection using Doppler radar based on physical characteristics of targets. IEEE Geosci. Remote Sens. Lett..

[11] Kim J.C., Jeong H.G., Lee S. (2021). Simultaneous Target Classification and Moving Direction Estimation in Millimeter-Wave Radar System. Sensors.

[12] Di Mattia V., Manfredi G., De Leo A., Russo P., Scalise L., Cerri G., Cardillo E. A feasibility study of a compact radar system for autonomous walking of blind people. Proceedings of the 2016 IEEE 2nd International Forum on Research and Technologies for Society and Industry Leveraging a Better Tomorrow (RTSI).

[13] Hyun E., Jin Y.S., Lee J.H. (2016). A pedestrian detection scheme using a coherent phase difference method based on 2D range-Doppler FMCW radar. Sensors.

[14] Ahmad W.A., Kucharski M., Ergintav A., Abouzaid S., Wessel J., Ng H.J., Kissinger D. (2020). Multimode W-Band and D-Band MIMO Scalable Radar Platform. IEEE Trans. Microw. Theory Tech..

[15] Swartzlander E.E., Saleh H.H. (2010). FFT implementation with fused floating-point operations. IEEE Trans. Comput..

[16] Chen J., Lei Y., Peng Y., He T., Deng Z. (2016). Configurable floating-point FFT accelerator on FPGA based multiple-rotation CORDIC. Chin. J. Electron..

[17] Chen X., Lei Y., Lu Z., Chen S. (2018). A variable-size FFT hardware accelerator based on matrix transposition. IEEE Trans. Very Large Scale Integr. (VLSI) Syst..

[18] Li Y., Chen H., Xie Y. (2021). An FPGA-Based Four-Channel 128k-Point FFT Processor Suitable for Spaceborne SAR. Electronics.

[19] Hou J., Zhu Y., Shen Y., Li M., Wu Q., Wu H. Enhancing precision and bandwidth in cloud computing: Implementation of a novel floating-point format on fpga. Proceedings of the 2017 IEEE 4th International Conference on Cyber Security and Cloud Computing (CSCloud).

[20] Kronauge M., Rohling H. (2013). Fast two-dimensional CFAR procedure. IEEE Trans. Aerosp. Electron. Syst..

[21] Zhang S.S., Zeng T., Long T., Yuan H.P. Dim target detection based on keystone transform. Proceedings of the IEEE International Radar Conference.

[22] Peng W. (2019). Decision-making Optimization of Logistics Supply Chain Based on Small Target Echo Coherent Accumulation Algorithm Based on LTE Signal. Acoust. Speech Signal Process..

[23] Zheng Q., Yang L., Xie Y., Li J., Hu T., Zhu J., Xu Z. (2020). A Target Detection Scheme with Decreased Complexity and Enhanced Performance for Range-Doppler FMCW Radar. IEEE Trans. Instrum. Meas..

[24] Hyun E., Jin Y.S., Lee J.H. (2017). Design and development of automotive blind spot detection radar system based on ROI pre-processing scheme. Int. J. Automot. Technol..

[25] Alizadeh M., Shaker G., De Almeida J.C.M., Morita P.P., Safavi-Naeini S. (2019). Remote monitoring of human vital signs using mm-Wave FMCW radar. IEEE Access.

[26] Wang Y., Wang W., Zhou M., Ren A., Tian Z. (2020). Remote monitoring of human vital signs based on 77-GHz mm-wave FMCW radar. Sensors.

[27] Garrido M., Qureshi F., Takala J., Gustafsson O. (2019). Hardware architectures for the fast Fourier transform. Handbook of Signal Processing Systems.

[28] Garrido M., Sánchez M.Á., López-Vallejo M.L., Grajal J. (2016). A 4096-point radix-4 memory-based FFT using DSP slices. IEEE Trans. Very Large Scale Integr. (VLSI) Syst..

[29] Liu S., Liu D. (2018). A high-flexible low-latency memory-based FFT processor for 4G, WLAN, and future 5G. IEEE Trans. Very Large Scale Integr. (VLSI) Syst..

[30] Hsiao C.F., Chen Y., Lee C.Y. (2010). A generalized mixed-radix algorithm for memory-based FFT processors. IEEE Trans. Circuits Syst. II Express Briefs.

[31] Jung Y., Cho J., Lee S., Jung Y. (2019). Area-efficient pipelined FFT processor for zero-padded signals. Electronics.

[32] Jeon H., Jung Y., Lee S., Jung Y. (2020). Area-Efficient Short-Time Fourier Transform Processor for Time–Frequency Analysis of Non-Stationary Signals. Appl. Sci..

[33] Hügler P., Geiger M., Waldschmidt C. RCS measurements of a human hand for radar-based gesture recognition at E-band. Proceedings of the 2016 German Microwave Conference (GeMiC).

[34] Kärnfelt C., Péden A., Bazzi A., Shhadé G.E.H., Abbas M., Chonavel T. 77 GHz ACC radar simulation platform. Proceedings of the 2009 9th International Conference on Intelligent Transport Systems Telecommunications (ITST).

[35] Kahan W. (1996). IEEE Standard 754 for Binary Floating-Point Arithmetic. Lect. Notes Status IEEE.

[36] Adjoudani A., Beck E.C., Burg A.P., Djuknic G.M., Gvoth T.G., Haessig D., Wolniansky P.W. (2003). Prototype experience for MIMO BLAST over third-generation wireless system. IEEE J. Sel. Areas Commun..

[37] Muller J.M. (1985). Discrete basis and computation of elementary functions. IEEE Trans. Comput..

[38] Yu J.Y., Huang D., Li X., Xu K., Guo L.M., Gao J.J. (2013). Four parallel channels radix-4 FFT with single floating-point butterfly. Appl. Mech. Mater..

[39] Xilinx, Inc.. https://www.xilinx.com/support/documentation/ip_documentation/xfft/v9_0/pg109-xfft.pdf.

[40] Gautam V., Ray K.C., Haddow P. Hardware efficient design of variable length FFT processor. Proceedings of the 14th IEEE International Symposium on Design and Diagnostics of Electronic Circuits and Systems.

[41] Wang C., Gan W.S., Jong C.C., Luo J. A low-cost 256-point FFT processor for portable speech and audio applications. Proceedings of the 2007 International Symposium on Integrated Circuits.

